# Strain-Specific Protective Effect of the Immunity Induced by Live Malarial Sporozoites under Chloroquine Cover

**DOI:** 10.1371/journal.pone.0045861

**Published:** 2012-09-24

**Authors:** Wathsala Wijayalath, Sandra Cheesman, Kazuyuki Tanabe, Shiroma Handunnetti, Richard Carter, Sisira Pathirana

**Affiliations:** 1 Malaria Research Unit, Department of Parasitology, Faculty of Medicine, University of Colombo, Colombo, Sri Lanka; 2 School of Biological Sciences, Institute of Immunology and Infection Research, University of Edinburgh, Edinburgh, Scotland, United Kingdom; 3 Department of Molecular Protozoology, Research Institute for Microbial Diseases, Osaka University, Suita, Osaka, Japan; 4 Institute of Biochemistry, Molecular Biology and Biotechnology, University of Colombo, Colombo, Sri Lanka; Technion-Israel Institute of Technology, Israel

## Abstract

The efficacy of a whole-sporozoite malaria vaccine would partly be determined by the strain-specificity of the protective responses against malarial sporozoites and liver-stage parasites. Evidence from previous reports were inconsistent, where some studies have shown that the protective immunity induced by irradiated or live sporozoites in rodents or humans were cross-protective and in others strain-specific. In the present work, we have studied the strain-specificity of live sporozoite-induced immunity using two genetically and immunologically different strains of *Plasmodium cynomolgi*, Pc746 and PcCeylon, in toque monkeys. Two groups of monkeys were immunized against live sporozoites of either the Pc746 (n = 5), or the PcCeylon (n = 4) strain, by the bites of 2–4 sporozoite-infected *Anopheles tessellates* mosquitoes per monkey under concurrent treatments with chloroquine and primaquine to abrogate detectable blood infections. Subsequently, a group of non-immunized monkeys (n = 4), and the two groups of immunized monkeys were challenged with a mixture of sporozoites of the two strains by the bites of 2–5 infective mosquitoes from each strain per monkey. In order to determine the strain-specificity of the protective immunity, the proportions of parasites of the two strains in the challenge infections were quantified using an allele quantification assay, Pyrosequencing™, based on a single nucleotide polymorphism (SNP) in the parasites’ circumsporozoite protein gene. The Pyrosequencing™ data showed that a significant reduction of parasites of the immunizing strain in each group of strain-specifically immunized monkeys had occurred, indicating a stronger killing effect on parasites of the immunizing strain. Thus, the protective immunity developed following a single, live sporozoite/chloroquine immunization, acted specifically against the immunizing strain and was, therefore, strain-specific. As our experiment does not allow us to determine the parasite stage at which the strain-specific protective immunity is directed, it is possible that the target of this immunity could be either the pre-erythrocytic stage, or the blood-stage, or both.

## Introduction

One of the approaches for developing an effective anti-malaria vaccine is to induce a protective immune response by vaccination with whole parasites [1]. Examples include vaccination with irradiated [2] or genetically attenuated sporozoites [3], with live sporozoite inoculation under a blood-schizonticidal drug cover [4], [5] or with attenuated asexual blood-stage parasites [6]. Indeed, whole sporozoite vaccination remains the most effective way to induce sterile immunity against malaria pre-erythrocytic (PE) stages [7]. This is probably, at least in part, because it exposes a host to a broad array of antigens expressed on the sporozoites and throughout liver-stage development.

The efficacy of a whole sporozoite vaccine, as of any other, will partly depend upon the degree of diversity of the genes that code for target antigens of vaccine-induced immunity [8]–[11]. This diversity may lead to parasite strain- or genotype-specific protective immune responses that would limit the degree of cross-protection to different parasite strains. Several studies have already addressed the strain-specificity of protective immune responses to sporozoites and the subsequent PE stages [12]–[16] using different approaches. Some have shown that protection induced by *P. falciparum* [12]–[14] and *P. vivax* [12] irradiated sporozoites in humans are not strain-specific. Experiments with *P. cynomolgi* in hepatocyte cultures [15] and *P. chabaudi* in mice [16] showed evidence for strain-specific responses against PE stages. In most of these studies involving animals or human subjects, the degree of strain-specific protection was determined by the parasitological response following homologous or heterologous challenge in the immunized host. In the natural host/parasite system we used here [17], we measured the proportions of the blood-stage parasites using Pyrosequencing™ (PSQ) (an allele quantification assay), following sporozoite-induced mixed-strain challenge, in toque monkeys (*Macaca sinica*) which were strain-specifically immunized with either of the two strains of *P. cynomolgi*. Our results indicate that strain-specific protective immunity had been induced by a single immunization with live *P. cynomolgi* sporozoites under the cover of drugs that prevented active blood infection.

## Materials and Methods

### Ethics Statement

The study protocol was approved by the Ethics Review Committee of the Faculty of Medicine, University of Colombo, Sri Lanka (Permit NO: EC/05/21). Permission to use captured monkeys was obtained from the Department of Wild Life Conservation, Sri Lanka (Permit no: WL/3/2/1/6/4). Monkeys used in the study were captured by a team that included the first and corresponding authors of this manuscript. The animals were screened to ensure that they were older than 1 year, their weight was between 1.5–3.0 kg, and to ensure that they were not pregnant before being retained in captivity. The study was carried out in strict accordance with the recommendations of the Ethics Review Committee and adhered to international guidelines on the use of non-human primates in research.

Monkeys were housed in an animal facility and maintained under standard housekeeping conditions [17] with restricted access from outside the unit. The animals were kept inside 1 m×0.6 m×0.8 m cages, as non-aggressive pairs of the opposite sex or of the same sex. A numerical code was used to identify the experimental monkeys, who could be distinguished by their sex, size or color painted abdomens. Monkeys were exposed to natural light during the day time. A balanced diet comprising fruits, vegetables, grains and starch were provided twice a day, and a continuous supply of clean water was available throughout the day via stainless steel cups fixed to the front facet of each cage. The monkeys were observed daily to identify any behavioral or clinical changes in their condition (e.g., signs of dizziness, vomiting, diarrhea or changes in the amount of food eaten). Monkeys that became unhealthy because of causes unrelated to malaria infection were discontinued from the study and veterinary care was provided for them. Malaria infected monkeys were treated with anti-malarial drugs upon termination of each infection. All experimental procedures on monkeys were carried out by trained and experienced personnel adhering to standard operating procedures and in a minimally invasive manner with or without anesthesia. Monkeys were restrained using a sliding partition of the cages, for experimental procedures such as ear-pricks, intramuscular injections, or oral administration of anti-malarial drugs were given. When necessary, restrained monkeys were anesthetized by administration of an intra-muscular preparation of Ketamin hydrochloride (Themis Chemicals Ltd, India and Vindas Chemical Industries, India) using a dosage of 10 mg/kg body weight. This short-acting anesthetic lasts for approximately 10 min, which is sufficient time to perform mosquito feeds or other invasive procedures. Oral suspensions of anti-malarial drugs, mixed with glucose, were administered by sterile plastic transfer pipettes (BIO-RAD laboratories) to the restrained monkeys. Steel feeders were avoided to prevent oral injuries. All the experimental monkeys were drug-cured with chloroquine and primaquine and released back into their natural habitat upon completion of the experiment as per the recommendations of the Department of Wild Life Conservation and Ethics Review Committee [17]. Before release into the wild, certification from the Medical Research Institute, Ministry of Health, Sri Lanka, was provided to the Department of Wild Life Conservation certifying that the monkeys were malaria free. The released monkeys were considered unlikely to be harmful to the wild-life as they were considered free of laboratory acquired microorganisms as they were maintained in an animal facility with high standards of hygiene (i.e., all procedures had been conducted using sterile consumables).

### Parasites and Animals

The genetically and immunologically different strains of *Plasmodium cynomolgi*, PcCeylon and Pc746 strains, which were described previously, were used in the study [17], [18]. Selection of malaria free toque monkeys and allocation of monkeys in the study were carried out as described in our previous study [17]. Briefly, monkeys captured from the wild for the study were quarantined and monitored for: 1) current malaria infections by examining blood smears and, 2) the presence of anti-malarial antibodies by IFA. Malaria-free (i.e., negative blood smear and negative for anti-malaria parasite antibodies), mixed-age monkeys with body weights between 1·5–3·0 kg were selected. At least 4 weeks before the experiment commenced, all of the monkeys were treated orally with a 3·75-mg dose of primaquine phosphate (Atlantic Laboratories Corp. Ltd, Bangkok, Thailand) to eliminate any existing liver stage parasites.

### Infective Mosquitoes

The laboratory-bred *Anopheles tessellates* mosquito colony has been maintained in the insectary of the Malaria Research Unit [18], [19]. Batches of approximately 250 three-day old female *An. Tessellates* mosquitoes were fed each day on either a Pc746- or PcCeylon-infected, anesthetized donor monkey for 10–15 minutes during the early phase of blood infections (from day 3 of patency until the day of peak parasitaemia). Fed mosquitoes were kept in the insectary under controlled temperature (27°C) and humidity conditions (80%) with a continuous supply of a 4% glucose solution. Seven to nine and 12–14 days after the blood meal, 10–15 fed mosquitoes were randomly collected from each batch, dissected and their mid-guts and salivary glands were examined for the presence of oocysts and sporozoites respectively. Mosquitoes were considered infective when they had ≥10^2^ motile sporozoites in their salivary glands. The percentage of infective mosquitoes was calculated as (number of infective mosquitoes/total number of mosquitoes dissected)×100.

### Malaria Infections in the Monkeys

From a batch of mosquitoes in which more than 50% were sporozoite-infected, five mosquitoes were randomly collected and allowed to feed on an anesthetized experimental monkey. Fed mosquitoes were confirmed by observing their blood gorged abdomens and unfed mosquitoes were replaced by new ones. All the fed mosquitoes were dissected immediately after the blood meal to examine their salivary glands. The number of infective mosquitoes fed on each experimental monkey was confirmed by the presence of motile sporozoites within their salivary glands. If pre-defined numbers were not achieved, new mosquitoes were fed on each monkey one at a time until the required number of infective mosquitoes was obtained.

### Sporozoite-induced Infections in Non-immunized Control Monkeys

Sporozoite-induced single-strain infections of either PcCeylon or Pc746 were obtained in two groups of monkeys, 4 monkeys in each, via the bites of 2–6 infective mosquitoes. Bites from 2 to 6 infective mosquitoes from each of the two strains were simultaneously given to 7 monkeys to obtain sporozoite-induced mixed-strain infections. Asexual parasitaemias were recorded up to 28 days following the infective bites.

### Immunization and Challenge Infections

Two groups of malaria free toque monkeys were immunized with bites from 2–4 sporozoite infected mosquitoes of either the Pc746 (n = 5) or PcCeylon (n = 4) strain under chloroquine [20] and primaquine treatment. A 6 day chloroquine regimen was given intramuscularly to each monkey at a dosage of 10 mg/Kg bodyweight/day, starting on day 4 of the infective bites. A 5 day regimen of filter-sterilized primaquine, dissolved in water with glucose (to make a suspension of sweeter taste that would be easily swallowed by the monkeys) was given orally at a dosage of 3.75 mg/day/monkey, beginning on 27 and 25 days after PcCeylon and Pc746 infective bites, respectively. Four non-immunized control monkeys were also given a simultaneous course of chloroquine and primaquine. Daily blood smears were examined for malaria parasites [17] for a 1 month period following the drug treatments. Immunization against the PcCeylon strain was delayed by 42 days compared to Pc746 due to practical difficulties in obtaining infective mosquitoes simultaneously. Following the immunizations, all three groups were challenged simultaneously, with a mixture of sporozoites of both strains, by giving bites from 2–5 sporozoite-infected mosquitoes from each strain per monkey. Thus, PcCeylon- and Pc746-immunized monkeys were challenged at 100 and 140 days respectively after immunization, which were 73 and 115 days respectively after primaquine treatment. Asexual parasitaemias were recorded [17] up to 23 days post-challenge. Once the infections reached ∼0.03% asexual parasitaemia, blood samples (10–20 µl) were collected for 4–8 consecutive days, for Pyrosequencing™ (PSQ) analysis. This was achieved by direct sampling of blood on to Whatman FTA cards from the marginal ear vein of the infected monkeys.

### PSQ Assays

PSQ assays were conducted to quantify the proportions of the two strains in the mixed-strain challenge infections, based on a SNP in the *csp* gene of the two *P. cynomolgi* strains. Nearly complete *csp* sequences were obtained from genomic DNA of the PcCeylon and Pc746 strains by direct sequencing of PCR products using two primers; Pc*csp*-F1, 5′- ATACAAGAACAAGATGAAGAACTTA-3′and Pc*csp*-R1, 5′-TGTCAGCTACTTAATTGAATAATGCTA-3′. Detailed procedures for PCR and sequencing have been described previously [21]. The two *csp* sequences are available in DDBJ/EMBL/GenBank under the accession numbers AB524341- AB524342. The SNP within the *csp* gene used for the PSQ assay was located at the C terminal non-repeat region of the *csp* gene and maps to the nucleotide position 1085 (C) of *csp* gene of PcCeylon and to position 1013 (T) of Pc746 strain. Assay Design Software selected a pair of primers to co-amplify 88 bp of the C terminal non-repeat region of the *csp* gene, containing the selected SNP. There were no other genetic differences found within this region. The PSQ primer was designed to bind to a region of the co-amplified PCR product that is also completely conserved between the two strains. Therefore, the primers used in the PSQ assay were expected to have similar binding affinities and extension kinetics with respect to the two selected *P. cynomolgi* sequences in mixed-strain infections. Sequences of the PCR and PSQ primers are as follows: Forward 5′-AATGCAGCTAACAAAAAACCAGAA-3′ (unmodified), Reverse 5′-TAAATATACCAGCGCACTTATCCA-3′(5′ biotinylated) and sequencing primer 5′ AAACCAGAAGAGCTTGAT 3′ (unmodified) (Eurogentec). Genomic DNA was purified from the disk punched from the Whatman FTA card that contained the infected blood spots after repeated washing steps as per the manufacturer’s instructions. PCR reagents were added to a 0.2 ml PCR tube containing the disk with the purified DNA. The conditions used for the PCR amplifications were as described previously [17]. Two PCR amplifications were carried out on 2 different disk punches obtained from blood spots derived from the same sample. PSQ™ assays were conducted according to the manufacturer’s protocols (Biotage) using a PSQ™ HS-96A instrument. Ten microlitres of biotinylated PCR product was used in each PSQ reaction following the conditions described previously [22]. For each sample, up to 3 repeated PSQ measurements were carried out on the same PCR product to quantify the proportions of each parasite strain. A 0.03% parasitaemia cut-off limit was chosen as the limits of detection because at parasitaemias lower than this value, quantitation of the SNP proportions using PSQ was less accurate.

### Statistical Analysis

A Mann-Whitney U test for two independent variables was used for comparisons between the groups at a 95% confidence interval (SPSS statistical software, standard version release 15.0).

## Results

Malaria-free toque monkeys were immunized by the bites of *An. tessellates* mosquitoes infected with live sporozoites of either of the two *P. cynomolgi* strains, Pc746 or PcCeylon. To prevent either an active primary blood infection or blood infection due to relapse from hypnozoites in the liver, these infective bites were delivered by concurrent treatment with the blood-schizonticide, chloroquine, and subsequently with primaquine to eliminate hypnozoites, as described in the Materials and Methods. Absence of detectable parasitaemia in the thick blood smears following chloroquine and primaquine treatments confirmed that these treatments were effective in abrogating detectable primary and relapse blood infections in the sporozoite-infected monkeys. In contrast, chloroquine untreated controls who received bites from a comparable number of infective mosquitoes carrying either of the two strains of *P. cynomolgi* developed primary blood infections (Table 1, PcCeylon and Pc746 single-strain infections). Thus, the sporozoite-infected, drug-treated monkeys should also have developed PE stages to their maturity to the point of releasing infective merozoites into the blood.

**Table 1:** Asexual parasitaemia of sporozoite-induced infections in non-immunized monkeys.

Group	Pre-patent Period^a^	% Peak parasitaemia	Day of peak parasitaemia^b^	% total parasitaemia^c^
PcCeylon single-strain infections (n = 4)	12.5±1.0	0.18±0.2	21.0±5.2	0.29±0.2
Pc746 single-strain infections (n = 4)	12.5±1.2	0.19±0.2	18.8±0.5	0.36±0.3
Mixed-strain infections (n = 7)	12.6±1.1	0.27±0.2	19.0±2.4	0.59±0.4

a Days until detectable parasitaemia in the thick blood smear from the day of sporozoite inoculation.

b Days until peak asexual parasitaemia from the day of sporozoite inoculation.

c Sum of the asexual parasitaemia for eight consecutive days from the day of detectable parasitaemia in a thick blood smear.

There were no significant differences between the three types of infections; P>0.05.

To determine the strain-specific protective effect of the live sporozoite-induced immunity under drug cover, we challenged both non-immunized monkeys and strain-specifically immunized monkeys with a mixture of infective bites of both strains as described in Materials and Methods. The ensuing blood infections were microscopically monitored for asexual parasitaemia and the proportions of the individual strains in those infections were quantified by the PSQ assay. PSQ values were recorded over 4–8 consecutive days starting from between 15 and 18 days post- challenge (starting from and including the day on which a monkey first reached or passed 0.03% parasitaemia). The day of the first such record in each monkey is designated “Day 1” hereinafter in the text.

**Figure 1:**
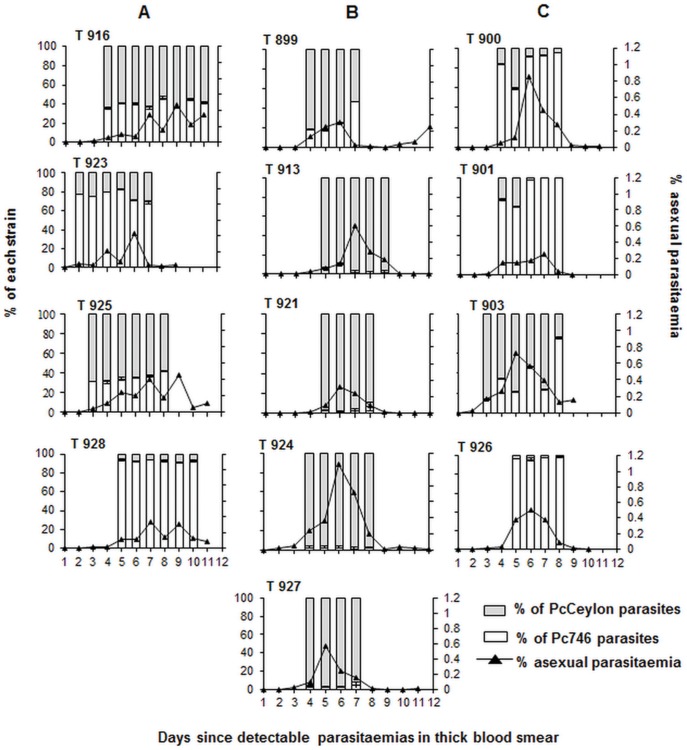
PSQ assay based on a SNP within the *csp* gene detects the strain-specific protective effect. Toque monkeys were strain-specifically immunized with live sporozoites (by infective mosquito bites) of Pc746 and PcCeylon strains under chloroquine and challenged with a mixture of bites of PcCeylon and Pc746 live sporozoite-infected mosquitoes. Open portions of columns represent Pc746 proportions and shaded portions of columns represent PcCeylon proportions in challenge infections of (A) non-immunized, n = 4 (B) Pc746 immunized, n = 5 and (C) PcCeylon immunized monkeys, n = 4. The absolute asexual parasitaemia, as a percent of red blood cells infected, are indicated by the lines connecting filled triangles. Error bars represent the standard deviation between the 3 to 6 repeated PSQ measurements of each monkey represented in each experimental condition. The proportion of parasites of each strain was recorded for 4 to 8 consecutive days starting from and including the day on which a monkey first reached or passed 0.03% asexual parasitaemia.The day of the first such record in each monkey is designated “Day 1” in the text. The Mann-Whitney U test for two independent variables (at 95% confidence level) was used to compare the proportions of Pc746 in Pc746 immunized and PcCeylon immunized groups on each day from “Day 1” to “Day 4”. P = 0.027 for “Day 1”, “Day 3”, and “Day 4”; P = 0.014 for “Day 2”.

Non-immunized control monkeys infected with sporozoites of a single strain, either PcCeylon or Pc746, and also of mixed-strain infections the two strains, had comparable characteristics in the resulting blood infections as regards to peak parasitaemia and time to peak parasitaemia (Table 1). In mixed-strain infections of the four non-immunized monkeys, the proportions in the blood of Pc746 and PcCeylon, as measured by the PSQ assay, remained remarkably constant throughout the period of measurements from “Day 1” to “Day 6” or “8” (Figure 1A). This shows that in sporozoite-induced mixed-strain infections in malaria non-immune monkeys, neither Pc746 nor PcCeylon outgrows the other.

Following the mixed-strain challenge with sporozoites, the blood infections in the Pc746 and PcCeylon immunized monkeys, tended to have similar characteristics (Figure 1B and 1C, Table 2) distinct from those in the non-immunized (Figure 1A and Table 2). In the immunized monkeys, peak parasitaemias were higher and tended to be passed a few days earlier than in the non-immunized animals, and significantly so in the case of the Pc746 immunized monkeys (cf Figure 1B and 1C with 1A, Table 2). Likewise, by day 9 post-patency following the mixed-strain sporozoite challenge, asexual parasitaemia in both Pc746-immunized (0.043%±0.079; P = 0.027) and PcCeylon-immunized (0.048%±0.07; P = 0.043) monkeys had declined to levels significantly lower than those in the non-immunized animals (0.319%±0.199) (cf Figure 1A with 1B and 1C). This rapid clearance of the blood-stage parasites by the immunized monkeys indicates development of cross-protection against blood-stage parasites of both strains. The parasitaemia data does not, however, allow us to directly determine the strain-specific nature of the immune response.

**Table 2:** Course of asexual parasitaemia in sporozoite-induced mixed-strain challenge infections.

Group		Pre-patent Period^a^	Day of peak Parasitaemia^b^	% peak parasitaemia	% Total parasitaemia^c^
Pc746 immunized monkeys (n = 5)		11.2±0.4	17.2±0.4	0.6±0.3	1.3±0.8
PcCeylon immunized monkeys (n = 4)		12.0±1.4	19.0±1.6	0.5±0.2	1.4±0.6
Non- immunized monkeys (n = 4)		12.5±1.0	20.8±1.2	0.4±0.1	1.2±0.3
Pc746 vs PcCeylon immunized	P	0.3	0.06	1.0	0.5
Pc746 immunized vs Non immunized	P	0.02 *	0.01 *	0.6	0.8
PcCeylon immunized vs non-immunized	P	0.3	0.1	0.2	0.3

a Days until detectable parasitaemia in the thick blood smear from day of sporozoite- induced mixed-strain challenge.

b Days until peak asexual parasitaemia from day of sporozoite-induced mixed-strain challenge.

c Sum of the asexual parasitaemia for nine consecutive days from the day of detectable parasitaemia in a thick blood smear.

P P value of statistical significance by Mann-Whitney U test for two independent variables. The P value for each parameter is underlined.

* Statistically significant.

The proportions of Pc746 parasites, as measured by the PSQ assay (see Materials and Methods) in Pc746-immunized monkeys, were significantly lower than their proportions in PcCeylon-immunized monkeys from the earliest time of measurement (“Day 1”) and this remained true for all of the remaining days of measurement (cf Figure 1B and 1C) (P = 0.027 for “Day 1”, “Day 3” and “Day 4”; P = 0.014 for “Day 2”). The reciprocal was true for the proportions of PcCeylon parasites in monkeys immunized with either strain (cf Figure 1B and 1C) (P = 0.027 for “Day 1”, “Day 3” and “Day 4”; P = 0.014 for “Day 2”). These results indicate that the parasite killing effect of the immunizations was directed primarily against the immunizing, or homologous, strain in a mixed strain sporozoite challenge infection. This had already come into effect by the time of the first PSQ measurements (days 15–18 post-challenge) of the blood infections following the mixed-strain sporozoite challenges.

In Pc746-immunized monkeys, the strength of the homologous strain killing was so strong that Pc746 blood parasites were, in most monkeys, virtually absent throughout the days of measurement by the PSQ assay (Figure 1B). In the PcCeylon immunized monkeys, however, in which the proportions of the homologous, PcCeylon, parasites were still relatively high on “Day 1”, these homologous, PcCeylon strain parasites, tended to decline over the period of measurement (Figure 1C). This result suggests that at least part of the strain-specific parasite killing effects were acting against the blood-stage parasites themselves during the period of the patent infections.

## Discussion

The present study demonstrates that immunity developed following a single immunization with live sporozoite-infected mosquito bites of the two *P. cynomolgi* strains, PcCeylon and Pc746, in toque monkeys, under chloroquine and subsequent primaquine treatment, is largely specific to the immunizing strain of parasite. The effect was revealed by PSQ analysis of a SNP in the *csp* gene of *P. cynomolgi*, which allowed quantification of the proportions of the blood-stage parasites of two strains, PcCeylon and Pc746, in mixed-strain sporozoite challenge infections in the immunized monkeys. In both PcCeylon and Pc746 immunized monkeys, the preferential reduction of the strain homologous to the immunizing strain had largely (Pc746) or partly (PcCeylon) taken place before the first PSQ measurements that were made 15 to 18 days post mixed-strain sporozoite challenge. While this could be consistent with immunity having acted against sporozoites and/or PE stages of the parasites, the effect could also have been operative partly, or even solely, in the blood infection for the reason that several days of development of the parasites in the blood had taken place by the time the first PSQ measurements in the challenge infections were made. It is, indeed, probable that anti blood-stage parasite effects account, at least in part, for the strain-specific immunity induced in these infections. This is because there was a tendency for the immunization-homologous strain to continue to decline over the measured period of the blood infections in PcCeylon immunized monkeys.

In theory, we might expect to see a lower level of anti-parasite protection in the immunized monkeys if an extended period of time had elapsed since the first exposure and without repeated exposure to the parasites. However in the present study, the Pc746 immunized monkeys had stronger strain-specific protection than the PcCeylon immunized monkeys, regardless of the 42 day delay in immunizing the latter group (which had a shorter time period between intial exposure to the parasite and the challenge infection). The current study lacks direct evidence to explain the stronger strain-specific protective effect seen in Pc746 immunized monkeys than that oberved in the PcCeylon immunized group. Several possibilities exist that could account for this; for example, the Pc746 strain might simply induce stronger strain-specific immunity than the PcCeylon strain, or the strength of the strain-specific immunity in the two immunized groups might vary because of differences in the expression of immunodominant strain-specific antigenic epitopes between the two strains.

We also noted a pan-specific effect against blood-stage parasites, as there was an early clearance of blood-stage parasitaemia in the immunized monkeys. Both pan-specific and strain-specific effects of the immunity against the blood-stages could have been induced as the result of the release of the liver-stage merozoites into the blood during the immunizing infection. Since chloroquine has no effect on the development of the parasites in the liver, it does not prevent a first generation of merozoites from the liver invading the blood [4], [23]. Moreover, liver-stage merozoites express the same repertoire of antigens as do the blood stage merozoites, including the merozoite surface protein-1 (MSP1) antigen, which has been shown to be the major target of strain-specific parasite-killing immunity against blood stage parasites [24]. In *P. vivax*, MSP1 expressed during liver schizogony was shown to induce antibodies against it in human infections [25]. Such antibodies could, therefore, account for the strain- and pan-specific immune effects against blood-stage parasites following sporozoite-induced immunity.

Our results also show that a single live sporozoite infection of *P. cynomolgi* under chloroquine and primaquine cover is sufficient to elicit a significant strain-specific protective immune response (though not sterile) against challenge with sporozoites of *P. cynomolgi*. The strain-specific protective immunity induced in this manner persisted for 3 or 5 months, with PcCeylon and Pc746 respectively, in the absence of any PE stages, which were eliminated by primaquine treatment as described in Materials and Methods. Thus, it indicates that once the immunity is developed, persistence of liver-stages is not essential to maintain the immunity induced by live sporozoites under drug cover in this host-parasite system of malaria. While our present results do not allow us to determine the parasite stage against which the sporozoite-induced strain-specific immunity was acting, a genetic approach, linkage group selection (LGS) [24], is now available, which could allow the genes and antigens involved, and hence possibly the parasite stage against which the immunity is acting, to be identified.
